# Tetra­ethyl­ammonium trichlorido(η^6^-*p*-cymene)ruthenate(II)

**DOI:** 10.1107/S1600536809050168

**Published:** 2009-11-28

**Authors:** Fang-Hui Wu, Lude Lu, Taike Duan, Qian-Feng Zhang

**Affiliations:** a Materials Chemistry Laboratory, Nanjing University of Science and Technology, Nanjing 210094, People’s Republic of China; b Institute of Molecular Engineering and Applied Chemistry, Anhui University of Technology, Ma’anshan, Anhui 243002, People’s Republic of China

## Abstract

In the title salt, [(C_2_H_5_)_4_N][RuCl_3_(C_10_H_14_)], the Ru^II^ atom shows an octa­hedral coordination in which the aromatic ring of the *p*-cymene mol­ecule occupies three coordination positions.

## Related literature

For bond distances in the [Et_4_N]^+^ cation, see: Allen *et al.* (1987 ▶). For related structures, see: Arslan *et al.* (2009*a* ▶,*b*) ▶; Solari *et al.* (2007 ▶); Vock & Dyson (2007 ▶); Lalrempuia *et al.* (2005 ▶); Liu *et al.* (2004 ▶). For the applications of dinuclear [Ru(η^6^-arene)Cl_2_]_2_ complexes as precursors in inorganic synthesis, see: Le Bozec *et al.* (1989 ▶); Quebatte *et al.* (2005 ▶).
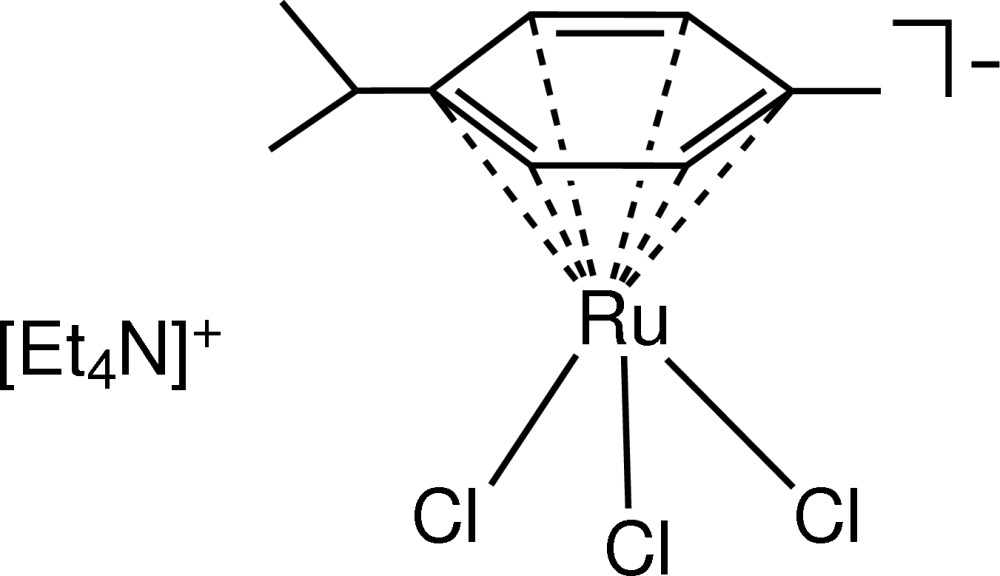


## Experimental

### 

#### Crystal data

(C_8_H_20_N)[RuCl_3_(C_10_H_14_)]
*M* *_r_* = 471.88
Monoclinic, 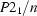

*a* = 9.5840 (1) Å
*b* = 22.3797 (2) Å
*c* = 10.2071 (1) Å
β = 98.668 (1)°
*V* = 2164.28 (4) Å^3^
*Z* = 4
Mo *K*α radiation
μ = 1.09 mm^−1^
*T* = 296 K
0.43 × 0.25 × 0.20 mm

#### Data collection

Bruker SMART CCD area-detector diffractometer
Absorption correction: multi-scan (*SADABS*; Sheldrick, 1996 ▶) *T*_min_ = 0.650, *T*_max_ = 0.811
21600 measured reflections
4985 independent reflections
4341 reflections with *I* > 2σ(*I*)
*R*_int_ = 0.021

#### Refinement

*R*[*F* ^2^ > 2σ(*F* ^2^)] = 0.023
*wR*(*F* ^2^) = 0.056
*S* = 1.02
4985 reflections
215 parameters
H-atom parameters constrained
Δρ_max_ = 0.38 e Å^−3^
Δρ_min_ = −0.29 e Å^−3^

### 

Data collection: *SMART* (Bruker, 1998 ▶); cell refinement: *SAINT-Plus* (Bruker, 1998 ▶); data reduction: *SAINT-Plus*; program(s) used to solve structure: *SHELXS97* (Sheldrick, 2008 ▶); program(s) used to refine structure: *SHELXL97* (Sheldrick, 2008 ▶); molecular graphics: *SHELXTL* (Sheldrick, 2008 ▶); software used to prepare material for publication: *SHELXTL*.

## Supplementary Material

Crystal structure: contains datablocks I, global. DOI: 10.1107/S1600536809050168/ng2680sup1.cif

Structure factors: contains datablocks I. DOI: 10.1107/S1600536809050168/ng2680Isup2.hkl

Additional supplementary materials:  crystallographic information; 3D view; checkCIF report

## Figures and Tables

**Table:** 

Ru1—Cl3	2.4216 (5)
Ru1—Cl2	2.4238 (5)
Ru1—Cl1	2.4381 (5)

**Table:** 

Cl3—Ru1—Cl2	87.805 (18)
Cl3—Ru1—Cl1	87.763 (19)
Cl2—Ru1—Cl1	87.35 (2)

**Fig. 1.**
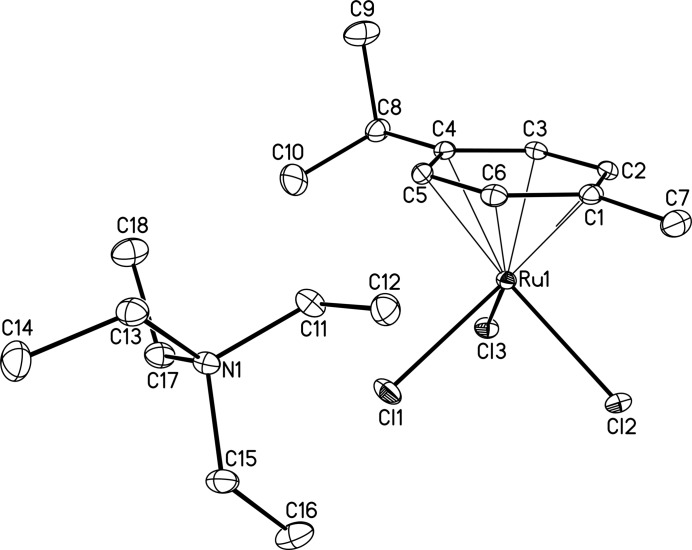
The structure of the title compound, showing the atom-numbering scheme and displacement ellipsoids at the 50% probability level.

## supplementary crystallographic information

### Comment

The general type dinuclear complexes [Ru(η^6^-arene)Cl_2_]_2_ are well established as useful synthetic precursors in preparative inorganic chemistry (Le Bozec *et al.* 1989). For example, the commercial [(*p*-cymene)RuCl_2_]_2_ was found to display an exceptionally high activity for atom transfer radical addition reactions under mild conditions (Quebatte *et al.* 2005). Relative to wide investigation of the dinuclear neutral [Ru(η^6^-arene)Cl_2_]_2_ complexes, mononuclear anions of type [Ru(η^6^-arene)Cl_3_] have rarely been described, although the labile chloride ligands in the [Ru(η^6^-arene)Cl_3_]^-^ species can be substituted to result in formation of a series of new complexes with [Ru(η^6^-arene)] fragments (Lalrempuia *et al.* 2005). It has recently been noted that [Ph_4_P][(*p*-cymene)RuCl_3_] was obtained from the reaction of [(*p*-cymene)RuCl_2_]_2_ with two equivalents of [Ph_4_P]Cl in dichloromethane (Vock & Dyson, 2007). With this idea in mind, in order to isolate trichlororuthenate(II) anion as an effective starting material, we are interested to carry out the similar reaction of [(*p*-cymene)RuCl_2_]_2_ with [Et_4_N]Cl.H_2_O, the mononuclear compound [Et_4_N][(*p*-cymene)RuCl_3_] is thus prepared and structurally characterized. In this paper, the initial results of this work are reported.

Complex (I) crystallizes in the monoclinic crystal system, containing two independent ions: [Et_4_N]^+^ cation and [RuCl_3_(C_10_H_14_)]^-^ anion. The molecular structure of the title compound is depicted in Fig. 1. The coordination geometry of ruthenium is pseudo-octahedral, with an average Ru—Cl bond length is 2.4278 (5) Å and the average Cl—Ru—Cl bond angle is 87.64 (2)°, which are compared with those reported in other related trichlororuthenate(II) complexes such as [Ph_4_P][(*p*-cymene)RuCl_3_] (av. Ru—Cl = 2.4450 (11) (5) Å, av. Cl—Ru—Cl = 87.35 (5)°) (Vock & Dyson, 2007), [C_14_H_17_N_2_S_2_][(*p*-cymene)RuCl_3_] (C_14_H_17_N_2_S_2_ = 1,3-bis(thiophen-2-ylmethyl)3,4,5,6- tetrahydropyrimidinium) (av. Ru—Cl = 2.4268 (11) Å, av. Cl—Ru—Cl = 87.10 (3)°) (Arslan *et al.* 2009*a*) and [C_13_H_15_N_2_S_2_][(*p*-cymene)RuCl_3_] (C_13_H_15_N_2_S_2_ = 1,3-(2-thienylmethyl)-4,5-dihydroimidazolium) (av. Ru—Cl = 2.4301 (11) Å, av. Cl—Ru—Cl = 87.61 (4)°) (Arslan *et al.* 2009*b*). The ruthenium atom exhibits a distorted octahedral coordination with the benzene ring of the *p*-cymene group formally occupying three of coordination positions and three terminal chloride atoms completing the coordination sphere. The Ru—C(ring) distances span the range 2.1380 (17)—2.1994 (18) Å in the title compound and compare well with those found in other *p*-cymene-trichlororuthenate(II) compounds (Vock & Dyson, 2007; Arslan *et al.* 2009*a*, 2009*b*). The distance between the centroid of the *p*-cymene ring and ruthenium is 1.648 (2) Å, which is longer than that reported in other ruthenium compounds with three-chloride ligands (Liu *et al.*, 2004; Solari *et al.*, 2007). The [Et_4_N]^+^ cation in the title compound has its expected structure as well as normal distances and angles, which will not be discussed further (Allen *et al.*, 1987).

### Experimental

Treatment of [(*p*-cymene)RuCl_2_]_2_ with two equivalents of [Et_4_N]Cl.H_2_O in a mixed THF/CH_2_Cl_2_ (1:1) solvent afforded the orange solution. The mixture was stirred for 2 h at room temperature, and then Et_2_O (50 ml) was added slowly and the precipitate that formed was filtered off with suction, washed with Et_2_O (3 *x* 10 ml) and dried *in vacuo*, yielding an orange solid in 92%. Recrystallization from CH_2_Cl_2_/Et_2_O (1:5) gave orange block crystals. Anal. Calcd. for C_18_H_34_NCl_3_Ru: C, 45.81; H, 7.26; N, 2.97%. Found: C, 45.76; H, 7.23; N, 2.92%.

### Refinement

H atoms were positioned geometrically and refined using a riding model (including free rotation about the ethanol C—C bond), with C—H = 0.95–0.99 Å and with *U*_iso_(H) = 1.2 (1.5 for methyl groups) times *U*_eq_(C).

### Crystal data

**Table:**

(C_8_H_20_N)[RuCl_3_(C_10_H_14_)]	*F*(000) = 976
*M**_r_* = 471.88	*D*_x_ = 1.448 Mg m^−^^3^
Monoclinic, *P*2_1_/*n*	Mo *K*α radiation, λ = 0.71073 Å
Hall symbol: -P 2yn	Cell parameters from 9980 reflections
*a* = 9.5840 (1) Å	θ = 2.2–27.4°
*b* = 22.3797 (2) Å	µ = 1.09 mm^−^^1^
*c* = 10.2071 (1) Å	*T* = 296 K
β = 98.668 (1)°	Block, orange
*V* = 2164.28 (4) Å^3^	0.43 × 0.25 × 0.20 mm
*Z* = 4	

### Data collection

**Table:**

Bruker SMART CCD area-detector diffractometer	4985 independent reflections
Radiation source: fine-focus sealed tube	4341 reflections with *I* > 2σ(*I*)
graphite	*R*_int_ = 0.021
phi and ω scans	θ_max_ = 27.6°, θ_min_ = 1.8°
Absorption correction: multi-scan (*SADABS*; Sheldrick, 1996)	*h* = −12→12
*T*_min_ = 0.650, *T*_max_ = 0.811	*k* = −29→25
21600 measured reflections	*l* = −11→13

### Refinement

**Table:**

Refinement on *F*^2^	Primary atom site location: structure-invariant direct methods
Least-squares matrix: full	Secondary atom site location: difference Fourier map
*R*[*F*^2^ > 2σ(*F*^2^)] = 0.023	Hydrogen site location: inferred from neighbouring sites
*wR*(*F*^2^) = 0.056	H-atom parameters constrained
*S* = 1.02	*w* = 1/[σ^2^(*F*_o_^2^) + (0.0263*P*)^2^ + 0.6416*P*] where *P* = (*F*_o_^2^ + 2*F*_c_^2^)/3
4985 reflections	(Δ/σ)_max_ = 0.001
215 parameters	Δρ_max_ = 0.38 e Å^−^^3^
0 restraints	Δρ_min_ = −0.29 e Å^−^^3^

### Special details

**Table:**

Geometry. All e.s.d.'s (except the e.s.d. in the dihedral angle between two l.s. planes) are estimated using the full covariance matrix. The cell e.s.d.'s are taken into account individually in the estimation of e.s.d.'s in distances, angles and torsion angles; correlations between e.s.d.'s in cell parameters are only used when they are defined by crystal symmetry. An approximate (isotropic) treatment of cell e.s.d.'s is used for estimating e.s.d.'s involving l.s. planes.
Refinement. Refinement of *F*^2^ against ALL reflections. The weighted *R*-factor *wR* and goodness of fit *S* are based on *F*^2^, conventional *R*-factors *R* are based on *F*, with *F* set to zero for negative *F*^2^. The threshold expression of *F*^2^ > σ(*F*^2^) is used only for calculating *R*-factors(gt) *etc*. and is not relevant to the choice of reflections for refinement. *R*-factors based on *F*^2^ are statistically about twice as large as those based on *F*, and *R*- factors based on ALL data will be even larger.

### Fractional atomic coordinates and isotropic or equivalent isotropic displacement parameters (Å^2^)

**Table:**

	*x*	*y*	*z*	*U*_iso_*/*U*_eq_	
Ru1	0.825774 (14)	0.078698 (6)	0.112583 (14)	0.03365 (5)	
Cl1	0.77065 (6)	0.17844 (2)	0.18611 (7)	0.06579 (16)	
Cl2	0.93200 (5)	0.12669 (2)	−0.06104 (5)	0.04993 (12)	
Cl3	1.05200 (5)	0.08727 (2)	0.25339 (5)	0.04920 (12)	
N1	0.31839 (16)	0.23549 (7)	0.13845 (16)	0.0425 (3)	
C1	0.6908 (2)	0.02804 (8)	−0.04218 (19)	0.0441 (4)	
C2	0.8053 (2)	−0.00847 (8)	0.01545 (19)	0.0423 (4)	
H2	0.8615	−0.0275	−0.0386	0.051*	
C3	0.83385 (19)	−0.01594 (8)	0.15289 (19)	0.0410 (4)	
H3	0.9099	−0.0397	0.1883	0.049*	
C4	0.7509 (2)	0.01140 (8)	0.24034 (19)	0.0428 (4)	
C5	0.6385 (2)	0.04798 (9)	0.1819 (2)	0.0463 (4)	
H5	0.5831	0.0674	0.2360	0.056*	
C6	0.60824 (19)	0.05587 (9)	0.0431 (2)	0.0459 (4)	
H6	0.5326	0.0798	0.0077	0.055*	
C7	0.6659 (3)	0.03821 (12)	−0.1883 (2)	0.0685 (7)	
H7A	0.6319	0.0020	−0.2325	0.103*	
H7B	0.7526	0.0499	−0.2172	0.103*	
H7C	0.5969	0.0692	−0.2091	0.103*	
C8	0.7882 (3)	0.00190 (11)	0.3880 (2)	0.0610 (6)	
H8	0.8898	−0.0062	0.4062	0.073*	
C9	0.7137 (3)	−0.05322 (14)	0.4288 (3)	0.0890 (9)	
H9A	0.7473	−0.0621	0.5202	0.134*	
H9B	0.7326	−0.0864	0.3747	0.134*	
H9C	0.6138	−0.0460	0.4176	0.134*	
C10	0.7606 (5)	0.05526 (15)	0.4702 (3)	0.1227 (15)	
H10A	0.8053	0.0899	0.4395	0.184*	
H10B	0.7981	0.0480	0.5613	0.184*	
H10C	0.6607	0.0620	0.4620	0.184*	
C11	0.3550 (2)	0.18162 (9)	0.0607 (2)	0.0576 (5)	
H11A	0.4569	0.1793	0.0676	0.069*	
H11B	0.3237	0.1460	0.1021	0.069*	
C12	0.2929 (3)	0.18074 (11)	−0.0842 (2)	0.0627 (6)	
H12A	0.1919	0.1783	−0.0928	0.094*	
H12B	0.3283	0.1467	−0.1261	0.094*	
H12C	0.3190	0.2166	−0.1259	0.094*	
C13	0.1606 (2)	0.23458 (10)	0.1427 (2)	0.0572 (5)	
H13A	0.1115	0.2368	0.0525	0.069*	
H13B	0.1368	0.1965	0.1790	0.069*	
C14	0.1062 (3)	0.28363 (14)	0.2219 (3)	0.0836 (8)	
H14A	0.1545	0.2823	0.3114	0.125*	
H14B	0.0068	0.2783	0.2218	0.125*	
H14C	0.1227	0.3216	0.1832	0.125*	
C15	0.3564 (2)	0.29344 (9)	0.0761 (2)	0.0579 (5)	
H15A	0.2973	0.2974	−0.0094	0.069*	
H15B	0.3331	0.3262	0.1312	0.069*	
C16	0.5080 (3)	0.30030 (12)	0.0562 (3)	0.0790 (8)	
H16A	0.5672	0.3019	0.1408	0.118*	
H16B	0.5186	0.3365	0.0082	0.118*	
H16C	0.5349	0.2668	0.0067	0.118*	
C17	0.4038 (2)	0.23164 (10)	0.2766 (2)	0.0566 (5)	
H17A	0.5030	0.2290	0.2678	0.068*	
H17B	0.3906	0.2684	0.3234	0.068*	
C18	0.3678 (3)	0.17998 (12)	0.3599 (2)	0.0766 (8)	
H18A	0.2708	0.1829	0.3726	0.115*	
H18B	0.4274	0.1810	0.4444	0.115*	
H18C	0.3824	0.1431	0.3158	0.115*	

### Atomic displacement parameters (Å^2^)

**Table:**

	*U*^11^	*U*^22^	*U*^33^	*U*^12^	*U*^13^	*U*^23^
Ru1	0.03190 (8)	0.02950 (8)	0.03954 (8)	−0.00283 (5)	0.00533 (5)	−0.00182 (5)
Cl1	0.0585 (3)	0.0406 (3)	0.0989 (5)	0.0030 (2)	0.0137 (3)	−0.0210 (3)
Cl2	0.0508 (3)	0.0453 (3)	0.0540 (3)	−0.0087 (2)	0.0087 (2)	0.0129 (2)
Cl3	0.0441 (3)	0.0497 (3)	0.0499 (3)	−0.0087 (2)	−0.0057 (2)	−0.0015 (2)
N1	0.0421 (8)	0.0339 (8)	0.0505 (9)	−0.0038 (6)	0.0039 (7)	0.0003 (7)
C1	0.0442 (10)	0.0453 (10)	0.0416 (10)	−0.0154 (8)	0.0022 (8)	−0.0011 (8)
C2	0.0455 (10)	0.0344 (9)	0.0487 (11)	−0.0097 (8)	0.0131 (8)	−0.0094 (8)
C3	0.0425 (10)	0.0290 (9)	0.0506 (10)	−0.0036 (7)	0.0046 (8)	0.0001 (7)
C4	0.0476 (10)	0.0392 (10)	0.0427 (10)	−0.0125 (8)	0.0098 (8)	−0.0011 (8)
C5	0.0388 (10)	0.0467 (11)	0.0571 (12)	−0.0088 (8)	0.0193 (9)	−0.0090 (9)
C6	0.0317 (9)	0.0445 (10)	0.0598 (12)	−0.0040 (8)	0.0017 (8)	0.0009 (9)
C7	0.0750 (16)	0.0813 (17)	0.0453 (12)	−0.0296 (13)	−0.0041 (11)	0.0054 (11)
C8	0.0694 (15)	0.0704 (15)	0.0428 (11)	−0.0186 (12)	0.0074 (10)	0.0022 (10)
C9	0.107 (2)	0.093 (2)	0.0583 (15)	−0.0324 (17)	−0.0146 (15)	0.0351 (15)
C10	0.228 (5)	0.098 (2)	0.0498 (16)	−0.030 (3)	0.047 (2)	−0.0173 (16)
C11	0.0643 (14)	0.0416 (11)	0.0652 (14)	0.0036 (10)	0.0047 (11)	−0.0064 (10)
C12	0.0642 (14)	0.0671 (15)	0.0575 (13)	−0.0073 (11)	0.0116 (11)	−0.0101 (11)
C13	0.0472 (11)	0.0541 (12)	0.0696 (14)	−0.0064 (10)	0.0060 (10)	0.0012 (11)
C14	0.0657 (16)	0.098 (2)	0.093 (2)	0.0031 (15)	0.0288 (14)	−0.0178 (16)
C15	0.0601 (13)	0.0395 (11)	0.0749 (15)	−0.0028 (9)	0.0124 (11)	0.0111 (10)
C16	0.0641 (15)	0.0775 (18)	0.098 (2)	−0.0149 (13)	0.0197 (14)	0.0261 (15)
C17	0.0574 (13)	0.0534 (12)	0.0559 (12)	−0.0111 (10)	−0.0021 (10)	−0.0020 (10)
C18	0.0853 (18)	0.0824 (18)	0.0569 (14)	−0.0217 (14)	−0.0058 (13)	0.0169 (13)

### Geometric parameters (Å, °)

**Table:**

Ru1—C5	2.1390 (17)	C9—H9A	0.9600
Ru1—C3	2.1568 (17)	C9—H9B	0.9600
Ru1—C6	2.1598 (18)	C9—H9C	0.9600
Ru1—C4	2.1829 (18)	C10—H10A	0.9600
Ru1—C2	2.1837 (17)	C10—H10B	0.9600
Ru1—C1	2.1994 (18)	C10—H10C	0.9600
Ru1—Cl3	2.4216 (5)	C11—C12	1.509 (3)
Ru1—Cl2	2.4238 (5)	C11—H11A	0.9700
Ru1—Cl1	2.4381 (5)	C11—H11B	0.9700
N1—C15	1.513 (2)	C12—H12A	0.9600
N1—C11	1.513 (3)	C12—H12B	0.9600
N1—C13	1.520 (2)	C12—H12C	0.9600
N1—C17	1.522 (3)	C13—C14	1.503 (3)
C1—C6	1.407 (3)	C13—H13A	0.9700
C1—C2	1.423 (3)	C13—H13B	0.9700
C1—C7	1.492 (3)	C14—H14A	0.9600
C2—C3	1.398 (3)	C14—H14B	0.9600
C2—H2	0.9300	C14—H14C	0.9600
C3—C4	1.420 (3)	C15—C16	1.505 (3)
C3—H3	0.9300	C15—H15A	0.9700
C4—C5	1.412 (3)	C15—H15B	0.9700
C4—C8	1.511 (3)	C16—H16A	0.9600
C5—C6	1.413 (3)	C16—H16B	0.9600
C5—H5	0.9300	C16—H16C	0.9600
C6—H6	0.9300	C17—C18	1.506 (3)
C7—H7A	0.9600	C17—H17A	0.9700
C7—H7B	0.9600	C17—H17B	0.9700
C7—H7C	0.9600	C18—H18A	0.9600
C8—C10	1.505 (4)	C18—H18B	0.9600
C8—C9	1.515 (3)	C18—H18C	0.9600
C8—H8	0.9800		
			
C5—Ru1—C3	68.23 (7)	C1—C6—H6	119.5
C5—Ru1—C6	38.38 (8)	C5—C6—H6	119.5
C3—Ru1—C6	80.53 (7)	Ru1—C6—H6	130.5
C5—Ru1—C4	38.11 (8)	C1—C7—H7A	109.5
C3—Ru1—C4	38.20 (7)	C1—C7—H7B	109.5
C6—Ru1—C4	69.20 (7)	H7A—C7—H7B	109.5
C5—Ru1—C2	80.83 (7)	C1—C7—H7C	109.5
C3—Ru1—C2	37.58 (7)	H7A—C7—H7C	109.5
C6—Ru1—C2	67.89 (7)	H7B—C7—H7C	109.5
C4—Ru1—C2	68.89 (7)	C10—C8—C4	114.1 (2)
C5—Ru1—C1	68.90 (7)	C10—C8—C9	111.2 (2)
C3—Ru1—C1	68.36 (7)	C4—C8—C9	109.79 (18)
C6—Ru1—C1	37.66 (7)	C10—C8—H8	107.1
C4—Ru1—C1	82.12 (7)	C4—C8—H8	107.1
C2—Ru1—C1	37.87 (7)	C9—C8—H8	107.1
C5—Ru1—Cl3	123.36 (6)	C8—C9—H9A	109.5
C3—Ru1—Cl3	87.79 (5)	C8—C9—H9B	109.5
C6—Ru1—Cl3	161.29 (6)	H9A—C9—H9B	109.5
C4—Ru1—Cl3	92.61 (5)	C8—C9—H9C	109.5
C2—Ru1—Cl3	110.56 (5)	H9A—C9—H9C	109.5
C1—Ru1—Cl3	147.57 (6)	H9B—C9—H9C	109.5
C5—Ru1—Cl2	148.21 (6)	C8—C10—H10A	109.5
C3—Ru1—Cl2	124.59 (5)	C8—C10—H10B	109.5
C6—Ru1—Cl2	110.85 (6)	H10A—C10—H10B	109.5
C4—Ru1—Cl2	162.66 (5)	C8—C10—H10C	109.5
C2—Ru1—Cl2	94.75 (5)	H10A—C10—H10C	109.5
C1—Ru1—Cl2	88.26 (5)	H10B—C10—H10C	109.5
Cl3—Ru1—Cl2	87.805 (18)	C12—C11—N1	115.85 (18)
C5—Ru1—Cl1	87.77 (5)	C12—C11—H11A	108.3
C3—Ru1—Cl1	147.52 (5)	N1—C11—H11A	108.3
C6—Ru1—Cl1	94.26 (6)	C12—C11—H11B	108.3
C4—Ru1—Cl1	109.99 (5)	N1—C11—H11B	108.3
C2—Ru1—Cl1	161.60 (5)	H11A—C11—H11B	107.4
C1—Ru1—Cl1	124.18 (6)	C11—C12—H12A	109.5
Cl3—Ru1—Cl1	87.763 (19)	C11—C12—H12B	109.5
Cl2—Ru1—Cl1	87.35 (2)	H12A—C12—H12B	109.5
C15—N1—C11	111.91 (16)	C11—C12—H12C	109.5
C15—N1—C13	109.08 (15)	H12A—C12—H12C	109.5
C11—N1—C13	108.33 (15)	H12B—C12—H12C	109.5
C15—N1—C17	107.95 (15)	C14—C13—N1	115.65 (19)
C11—N1—C17	107.80 (16)	C14—C13—H13A	108.4
C13—N1—C17	111.80 (16)	N1—C13—H13A	108.4
C6—C1—C2	117.97 (17)	C14—C13—H13B	108.4
C6—C1—C7	122.1 (2)	N1—C13—H13B	108.4
C2—C1—C7	119.9 (2)	H13A—C13—H13B	107.4
C6—C1—Ru1	69.65 (10)	C13—C14—H14A	109.5
C2—C1—Ru1	70.46 (10)	C13—C14—H14B	109.5
C7—C1—Ru1	128.89 (14)	H14A—C14—H14B	109.5
C3—C2—C1	120.37 (17)	C13—C14—H14C	109.5
C3—C2—Ru1	70.17 (10)	H14A—C14—H14C	109.5
C1—C2—Ru1	71.66 (10)	H14B—C14—H14C	109.5
C3—C2—H2	119.8	C16—C15—N1	116.26 (18)
C1—C2—H2	119.8	C16—C15—H15A	108.2
Ru1—C2—H2	131.1	N1—C15—H15A	108.2
C2—C3—C4	122.40 (18)	C16—C15—H15B	108.2
C2—C3—Ru1	72.26 (10)	N1—C15—H15B	108.2
C4—C3—Ru1	71.90 (10)	H15A—C15—H15B	107.4
C2—C3—H3	118.8	C15—C16—H16A	109.5
C4—C3—H3	118.8	C15—C16—H16B	109.5
Ru1—C3—H3	129.7	H16A—C16—H16B	109.5
C5—C4—C3	116.60 (17)	C15—C16—H16C	109.5
C5—C4—C8	123.66 (18)	H16A—C16—H16C	109.5
C3—C4—C8	119.71 (19)	H16B—C16—H16C	109.5
C5—C4—Ru1	69.26 (10)	C18—C17—N1	115.44 (17)
C3—C4—Ru1	69.90 (10)	C18—C17—H17A	108.4
C8—C4—Ru1	130.04 (13)	N1—C17—H17A	108.4
C4—C5—C6	121.64 (17)	C18—C17—H17B	108.4
C4—C5—Ru1	72.63 (10)	N1—C17—H17B	108.4
C6—C5—Ru1	71.61 (10)	H17A—C17—H17B	107.5
C4—C5—H5	119.2	C17—C18—H18A	109.5
C6—C5—H5	119.2	C17—C18—H18B	109.5
Ru1—C5—H5	129.0	H18A—C18—H18B	109.5
C1—C6—C5	121.00 (18)	C17—C18—H18C	109.5
C1—C6—Ru1	72.70 (10)	H18A—C18—H18C	109.5
C5—C6—Ru1	70.02 (10)	H18B—C18—H18C	109.5
			
C5—Ru1—C1—C6	28.91 (12)	C2—Ru1—C4—C3	27.98 (11)
C3—Ru1—C1—C6	102.96 (12)	C1—Ru1—C4—C3	64.77 (12)
C4—Ru1—C1—C6	65.97 (12)	Cl3—Ru1—C4—C3	−83.08 (11)
C2—Ru1—C1—C6	131.49 (17)	Cl2—Ru1—C4—C3	7.9 (2)
Cl3—Ru1—C1—C6	148.37 (10)	Cl1—Ru1—C4—C3	−171.65 (10)
Cl2—Ru1—C1—C6	−128.49 (11)	C5—Ru1—C4—C8	−117.1 (2)
Cl1—Ru1—C1—C6	−42.89 (13)	C3—Ru1—C4—C8	112.5 (2)
C5—Ru1—C1—C2	−102.58 (12)	C6—Ru1—C4—C8	−146.1 (2)
C3—Ru1—C1—C2	−28.52 (11)	C2—Ru1—C4—C8	140.4 (2)
C6—Ru1—C1—C2	−131.49 (17)	C1—Ru1—C4—C8	177.2 (2)
C4—Ru1—C1—C2	−65.52 (11)	Cl3—Ru1—C4—C8	29.4 (2)
Cl3—Ru1—C1—C2	16.88 (16)	Cl2—Ru1—C4—C8	120.4 (2)
Cl2—Ru1—C1—C2	100.02 (10)	Cl1—Ru1—C4—C8	−59.2 (2)
Cl1—Ru1—C1—C2	−174.37 (9)	C3—C4—C5—C6	1.4 (3)
C5—Ru1—C1—C7	144.2 (2)	C8—C4—C5—C6	179.53 (18)
C3—Ru1—C1—C7	−141.7 (2)	Ru1—C4—C5—C6	54.47 (16)
C6—Ru1—C1—C7	115.3 (3)	C3—C4—C5—Ru1	−53.09 (14)
C4—Ru1—C1—C7	−178.7 (2)	C8—C4—C5—Ru1	125.06 (18)
C2—Ru1—C1—C7	−113.2 (3)	C3—Ru1—C5—C4	30.46 (11)
Cl3—Ru1—C1—C7	−96.3 (2)	C6—Ru1—C5—C4	133.10 (17)
Cl2—Ru1—C1—C7	−13.2 (2)	C2—Ru1—C5—C4	67.33 (11)
Cl1—Ru1—C1—C7	72.5 (2)	C1—Ru1—C5—C4	104.70 (12)
C6—C1—C2—C3	−0.2 (3)	Cl3—Ru1—C5—C4	−41.32 (12)
C7—C1—C2—C3	176.88 (17)	Cl2—Ru1—C5—C4	151.52 (10)
Ru1—C1—C2—C3	52.52 (15)	Cl1—Ru1—C5—C4	−127.16 (11)
C6—C1—C2—Ru1	−52.68 (15)	C3—Ru1—C5—C6	−102.64 (12)
C7—C1—C2—Ru1	124.36 (17)	C4—Ru1—C5—C6	−133.10 (17)
C5—Ru1—C2—C3	−66.02 (12)	C2—Ru1—C5—C6	−65.77 (12)
C6—Ru1—C2—C3	−103.69 (12)	C1—Ru1—C5—C6	−28.40 (11)
C4—Ru1—C2—C3	−28.40 (11)	Cl3—Ru1—C5—C6	−174.42 (9)
C1—Ru1—C2—C3	−133.30 (16)	Cl2—Ru1—C5—C6	18.41 (17)
Cl3—Ru1—C2—C3	56.28 (11)	Cl1—Ru1—C5—C6	99.74 (11)
Cl2—Ru1—C2—C3	145.71 (10)	C2—C1—C6—C5	0.3 (3)
Cl1—Ru1—C2—C3	−118.41 (17)	C7—C1—C6—C5	−176.69 (18)
C5—Ru1—C2—C1	67.27 (11)	Ru1—C1—C6—C5	−52.79 (16)
C3—Ru1—C2—C1	133.30 (16)	C2—C1—C6—Ru1	53.07 (14)
C6—Ru1—C2—C1	29.60 (11)	C7—C1—C6—Ru1	−123.90 (18)
C4—Ru1—C2—C1	104.89 (12)	C4—C5—C6—C1	−0.9 (3)
Cl3—Ru1—C2—C1	−170.43 (9)	Ru1—C5—C6—C1	54.01 (16)
Cl2—Ru1—C2—C1	−81.00 (10)	C4—C5—C6—Ru1	−54.94 (16)
Cl1—Ru1—C2—C1	14.9 (2)	C5—Ru1—C6—C1	−133.41 (17)
C1—C2—C3—C4	0.7 (3)	C3—Ru1—C6—C1	−66.68 (12)
Ru1—C2—C3—C4	53.89 (15)	C4—Ru1—C6—C1	−104.60 (12)
C1—C2—C3—Ru1	−53.20 (15)	C2—Ru1—C6—C1	−29.76 (11)
C5—Ru1—C3—C2	103.75 (13)	Cl3—Ru1—C6—C1	−118.74 (17)
C6—Ru1—C3—C2	65.86 (12)	Cl2—Ru1—C6—C1	56.84 (12)
C4—Ru1—C3—C2	134.14 (17)	Cl1—Ru1—C6—C1	145.63 (11)
C1—Ru1—C3—C2	28.73 (11)	C3—Ru1—C6—C5	66.73 (12)
Cl3—Ru1—C3—C2	−128.80 (11)	C4—Ru1—C6—C5	28.82 (11)
Cl2—Ru1—C3—C2	−43.00 (13)	C2—Ru1—C6—C5	103.65 (13)
Cl1—Ru1—C3—C2	148.87 (9)	C1—Ru1—C6—C5	133.41 (17)
C5—Ru1—C3—C4	−30.39 (11)	Cl3—Ru1—C6—C5	14.7 (2)
C6—Ru1—C3—C4	−68.28 (12)	Cl2—Ru1—C6—C5	−169.74 (10)
C2—Ru1—C3—C4	−134.14 (17)	Cl1—Ru1—C6—C5	−80.96 (11)
C1—Ru1—C3—C4	−105.41 (12)	C5—C4—C8—C10	−32.8 (3)
Cl3—Ru1—C3—C4	97.05 (11)	C3—C4—C8—C10	145.3 (2)
Cl2—Ru1—C3—C4	−177.15 (9)	Ru1—C4—C8—C10	57.4 (3)
Cl1—Ru1—C3—C4	14.72 (17)	C5—C4—C8—C9	92.9 (3)
C2—C3—C4—C5	−1.3 (3)	C3—C4—C8—C9	−89.0 (3)
Ru1—C3—C4—C5	52.77 (14)	Ru1—C4—C8—C9	−176.91 (19)
C2—C3—C4—C8	−179.49 (17)	C15—N1—C11—C12	−55.0 (2)
Ru1—C3—C4—C8	−125.45 (17)	C13—N1—C11—C12	65.3 (2)
C2—C3—C4—Ru1	−54.04 (15)	C17—N1—C11—C12	−173.51 (18)
C3—Ru1—C4—C5	−130.42 (17)	C15—N1—C13—C14	−60.7 (3)
C6—Ru1—C4—C5	−29.01 (11)	C11—N1—C13—C14	177.2 (2)
C2—Ru1—C4—C5	−102.45 (12)	C17—N1—C13—C14	58.6 (3)
C1—Ru1—C4—C5	−65.65 (12)	C11—N1—C15—C16	−56.5 (3)
Cl3—Ru1—C4—C5	146.49 (10)	C13—N1—C15—C16	−176.3 (2)
Cl2—Ru1—C4—C5	−122.52 (17)	C17—N1—C15—C16	62.0 (3)
Cl1—Ru1—C4—C5	57.93 (11)	C15—N1—C17—C18	173.4 (2)
C5—Ru1—C4—C3	130.42 (17)	C11—N1—C17—C18	−65.5 (3)
C6—Ru1—C4—C3	101.42 (12)	C13—N1—C17—C18	53.4 (3)
